# Stimulation‐Evoked Resonant Neural Activity in the Subthalamic Nucleus Is Modulated by Sleep

**DOI:** 10.1002/mds.30063

**Published:** 2024-11-19

**Authors:** Christoph Wiest, Thomas G. Simpson, Alek Pogosyan, Harutomo Hasegawa, Shenghong He, Fernando Rodriguez Plazas, Laura Wehmeyer, Sahar Yassine, Xuanjun Guo, Rahul Shah, Anca Merla, Andrea Perera, Ahmed Raslan, Andrew O'Keeffe, Michael G. Hart, Francesca Morgante, Erlick A. Pereira, Keyoumars Ashkan, Huiling Tan

**Affiliations:** ^1^ Medical Research Council Brain Network Dynamics Unit, Nuffield Department of Clinical Neurosciences University of Oxford Oxford United Kingdom; ^2^ Department of Neurosurgery King's College Hospital London United Kingdom; ^3^ St George's, University of London and St. George's University Hospitals NHS Foundation Trust, Neuroscience and Cell Biology Research Institute London United Kingdom

**Keywords:** deep brain stimulation, evoked resonant neural activity, local field potentials, Parkinson's disease, sleep, subthalamic nucleus

## Abstract

**Background:**

Deep brain stimulation is a treatment for advanced Parkinson's disease and currently tuned to target motor symptoms during daytime. Parkinson's disease is associated with multiple nocturnal symptoms such as akinesia, insomnia, and sleep fragmentation, which may require adjustments of stimulation during sleep for best treatment outcome.

**Objectives:**

There is a need for a robust biomarker to guide stimulation titration across sleep stages. This study aimed to investigate whether evoked resonant neural activity (ERNA) is modulated by sleep.

**Methods:**

We recorded local field potentials from the subthalamic nucleus of four Parkinson's patients with externalized electrodes while applying single stimulation pulses to investigate the effect of sleep on ERNA.

**Results:**

We found that ERNA features change with wakefulness and sleep stages and are correlated with canonical frequency bands and heart rate.

**Conclusions:**

Given that ERNA modulates with sleep, it could be used as a robust marker for automatic stimulation titration during sleep. © 2024 The Author(s). *Movement Disorders* published by Wiley Periodicals LLC on behalf of International Parkinson and Movement Disorder Society.

Sleep disturbances, including fragmented sleep and insomnia, are common in Parkinson's disease (PD).^1^ Deep brain stimulation (DBS) of the subthalamic nucleus (STN) is an effective therapy for PD, but DBS settings that are tuned to improve daytime motor function and deal with daytime motor fluctuation might not be optimal for sleep. Recently it has been suggested that reducing stimulation intensity during non‐rapid eye movement (NREM) sleep may increase low frequency activities (slow waves) during sleep and potentially improve sleep quality.^2^ In addition, β‐triggered adaptive DBS may need to adjust the β threshold to capture pathological activities during NREM sleep, as average β power is reduced during this sleep stage. These studies highlight the importance of decoding sleep stages to further improve the efficacy of DBS during sleep.^3^, ^4^ DBS has been found to evoke resonant neural activity (ERNA) in the STN and globus pallidus internus (GPi).^5^, ^6^, ^7^ This oscillatory response to stimulation has an especially prominent amplitude in the dorsal subregion of the STN, is associated with clinical outcomes, and is a promising biomarker for lead localization.^6^ Here, we report for the first time that ERNA tracks sleep onset and sleep stage transitions, which may enhance and simplify automatic DBS titration at night.

## Methods

### Consent, Regulatory Approval and Patient Selection

This protocol was approved by the Health Research Authority United Kingdom (UK) and the local Research Ethics Committee (IRAS: 46576). Four patients with idiopathic PD undergoing bilateral STN‐DBS surgery were recruited for local field potential (LFP) recording at King's College Hospital National Health Service (NHS) Foundation Trust, London or St. George's University Hospital NHS Foundation Trust, London. Written informed consent was obtained in line with the Declaration of the Principles of Helsinki. Patients were selected by an interdisciplinary team as described before.^8^ The average age at recording was 63 ± 3.34 years (mean ± standard error of the mean) with average disease duration of 17.25 ± 4.31 years. Clinical details are summarized in Supplementary Table S1.

### Surgery and Lead Localization

The surgical target was the STN. DBS systems from two companies were implanted: Medtronic Neurological Division (Minneapolis, MN) (octopolar directional leads, SenSight model 33005) or Boston Scientific (Marlborough, MA) (octopolar directional leads, Vercise model DB‐2202). Electrodes were implanted as described before,^8^ connected to temporary lead extensions and externalized through the temporal or frontal scalp.

### Stimulation and Data Recording

Recordings were performed *on* dopaminergic medication, 4 to 6 days postoperatively, when leads were externalized. Monopolar stimulation was delivered using a European conformity (CE)‐marked ISIS neurostimulator (Inomed Neurocare, Emmendingen, Germany) and referenced to a self‐adhesive electrode attached to the patients' back. Stimuli comprised symmetric, constant‐current, biphasic pulses (60 μs, negative phase first). LFPs were amplified and sampled at 4096 Hz in unipolar mode using a multichannel amplifier (TMSi Saga, TMSi International, Oldenzaal, The Netherlands), with common mode rejection (with each LFP recording channels being referenced to the average of all unipolar recording channels) and custom‐written recording software written in C++.

### Experimental Paradigm

We first performed contact testing by delivering 25 single pulses at 4 mA spaced 2 to 2.5 seconds apart to all LFP channels in sequence while recording from all other channels. The channel that elicited largest ERNA amplitudes was chosen for stimulation. We recorded LFPs during sleep and spontaneous naps.

Sleep recordings were conducted with patients 1 and 2 in the evening (between 6 pm and 10 pm). The two patients were lying on a recliner armchair and bed, respectively, in a dark room with minimal noise and encouraged to fall asleep naturally. Single stimulation pulses were delivered every 2 to 2.5 seconds with concomitant LFP, electroencephalography (EEG) (Fz, F3, Cz, C3, Pz, and P3), electro‐oculography (EOG) (horizontal and vertical), electrocardiography (ECG), electromyography (EMG) (submental), and accelerometer (upper and lower limb) recordings.

Nap recordings from patients 3 and 4 were conducted in the afternoon (between 2 pm and 5 pm) when patients were comfortably seated in an armchair. Patients fell asleep spontaneously without instructions and were woken up twice (patient 3) and once (patient 4). Single stimulation pulses were delivered every 2 to 2.5 seconds with concomitant LFP, and EEG (Cz, C3, C4, CPz, CP3, and CP4). All participants confirmed that they fell asleep during testing, which was consistent with clinical observation.

As N1 is generally difficult to distinguish from wakefulness and physiologically distinct from other NREM stages, and REM sleep was detected only briefly if at all because of our relatively short recordings, we focused our analysis on N2 and N3 stages versus wakefulness, as in a recent study.^9^

### Signal Processing

Data analysis was performed in MATLAB (version 2023b, The MathWorks, Natick, MA) and Python.

#### Signal Processing: LFP Analysis

Spectral power was estimated from the same contact used for recording ERNA, by applying continuous wavelet transform (10 wavelet cycles) for the following frequency bands: Δ (1–4 Hz), θ (5–7 Hz), α (8–12 Hz), ς (13–16 Hz) (also referred to as low‐β in some literature), β (13–30 Hz), and γ (31–40 Hz). Spectral power was normalized to the average power between 1 and 40 Hz.

**FIG. 1:**
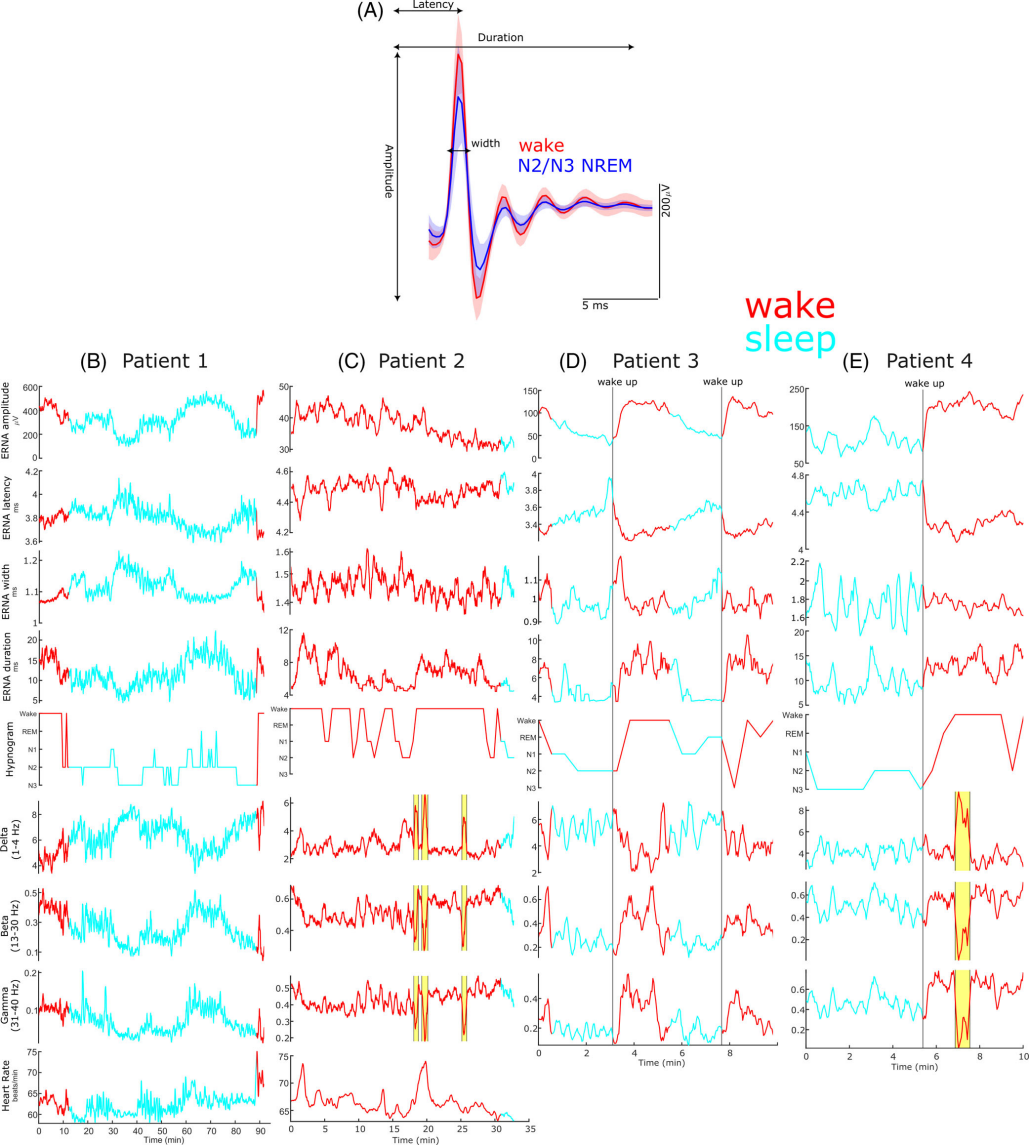
Evoked resonant neural activity (ERNA) and spectral local field potential (LFP) power modulation during sleep. (**A**) ERNA waveform during wakefulness and N2/N3 non‐rapid eye movement (NREM) sleep in patient 1 (mean ± standard deviation). (**B**–**E**) ERNA features, sleep stages, spectral LFP power bands and heart rate (in **B** + **C**) are shown during sleep/nap for patients 1 to 4. Yellow rectangles in (**C**) and (**E**) denote movement artefacts, which were excluded from the analysis. Vertical lines in (**D**) and (**E**) denote the moment when patients were woken up. In (**A**) red lines indicate awake stages and blue lines indicate N2/N3 sleep stages. In (**B**–**E**) red lines indicate awake stages and green lines indicate sleep. [Color figure can be viewed at wileyonlinelibrary.com]

ERNA features (amplitude, latency, duration, and width as shown in Fig. 1A) were extracted using similar methods as reported in previous studies^10^, ^11^ (more details in Supplementary Methods S1).

#### Signal Processing: Hypnograms

We removed stimulation artefacts in the EEG recordings and interpolated the missing signal. Subsequently, the YASA toolbox was used for sleep stage labeling,^12^ which outputs a classification for each 30‐second epoch. In patients 1 and 2, one EOG, a submental EMG, and one EEG contact were used as inputs for YASA. In patients 3 and 4, one single EEG channel was used as input (more details in the Supplementary Methods Data S1).

### Statistics

A Shapiro–Wilk test was used to assess normality and subsequently we performed a two‐sample *t* test or Wilcoxon rank sum test. When correlations are reported, we performed a Spearman rank correlation, and *P*‐values are reported after false discovery rate correction.

### Decoding Sleep Stages

Simple binary classifiers (logistic regression and support vector machine) were used to detect NREM sleep from wake using either ERNA features or spectral features extracted from STN LFPs. The approach to test the classification performance of these different features is described in Supplementary Methods Data S1.

## Results

### ERNA Is Modulated by Sleep Onset, Transition between Sleep Stages and Awakening

**FIG. 2:**
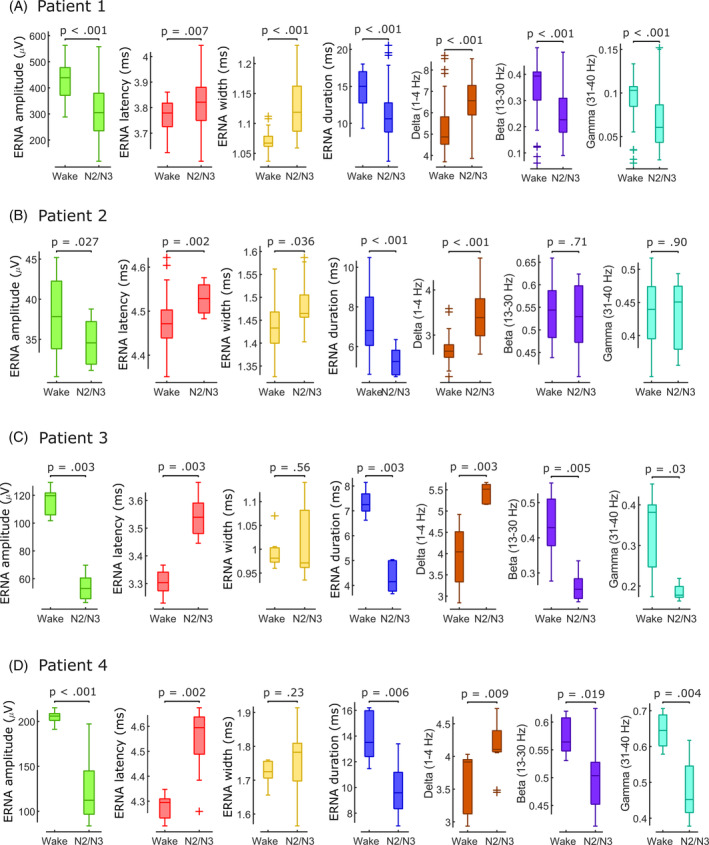
Evoked resonant neural activity (ERNA) and spectral local field potential (LFP) features differ between wakefulness and non‐rapid eye movement (NREM) sleep. (**A**–**D**) ERNA features and spectral LFP power bands change between wakefulness and N2/N3 NREM sleep in patients 1–4. [Color figure can be viewed at wileyonlinelibrary.com]

In patient 1, we recorded a full sleep cycle (Fig. 1B) and found that ERNA amplitude (*P* < 0.001; Cohen's *d* = 1.19) and duration (*P* < 0.001; *d* = 1.11) decrease from awake to N2/N3, whereas latency (*P* = 0.007; *d* = 0.56) and width (*P* < 0.001; *d* = 1.35) increase (Fig. 2A). ERNA amplitude and duration were positively correlated with θ to γ power, heart rate and sleep stages, with inverse relationships for latency and width (all *P* < 0.001).

In patient 2, we recorded the first ~33 minutes of a sleep cycle (Fig. 1C) and found similar ERNA changes: amplitude (*P* = 0.027; *d* = 0.80) and duration (*P* < 0.001; *d* = 1.32) decreased from awake to N2/N3, latency (*P* = 0.002; *d* = 0.99) and width (*P* = 0.036; *d* = 0.93) increased (Fig. 2B). Although Δ activity increased from awake to N2/N3 (*P* < 0.001; *d* = 2.00), classical β (*P* = 0.71; *d* = 0.19) and γ (*P* = 0.90; *d* = 0.07) power suppression was not observed in this patient manifesting as negative correlations between these frequencies and ERNA amplitude (all *P* < 0.001). This may explain why EEG‐based sleep stage identification did not yield consistent labelling.

In patients 3 and 4 (Fig. 1D,E), ERNA changed with the identified sleep stages: amplitude (patient 3: *P* = 0.003; *d* = 5.98; patient 4: *P* < 0.001; *d* = 2.70) and duration (patient 3: *P* = 0.003; *d* = 5.31; patient 4: *P* = 0.006; *d* = 1.97) decrease from awake to N2/N3 (Fig. 2C,D), whereas latency increases (patient 3: *P* = 0.003; *d* = 3.72; patient 4: *P* = 0.002; *d* = 2.50) and width remained unchanged (patient 3: *P* = 0.56; *d* = 0.36; patient 4: *P* = 0.23; *d* = 0.33). Positive relationships were observed between ERNA amplitude, duration and α to γ activity and negative relationships between ERNA amplitude, duration and Δ and θ power (all *P* < 0.001). Of note, cued awakenings resulted in immediate marked changes of ERNA (see Video S1) and spectral power supporting their modulation by the sleep/wake cycle.

### ERNA Is a Candidate for Classifying NREM Versus Wakefulness

We find ERNA‐derived features are promising candidates for sleep stage classification, where the amplitude achieves good performance on average (area under curve [AUC] = 97.2%, accuracy = 91.2%). Simple machine learning classification algorithms (support vector machine and logistic regression) using ERNA amplitude alone outperformed (AUC = +5.7%, accuracy = +4.1%) the model using a combination of spectral features (AUC = 91.5%, accuracy = 87.1%) for decoding NREM sleep versus wake (Supplementary Fig. S1).

## Discussion

This is the first study that shows STN ERNA modulation during sleep suggesting that ERNA is a promising candidate for sleep stage classification.

### Mechanism Underlying ERNA Modulation during Sleep

ERNA has been suggested to result from inhibitory‐excitatory reciprocal connections between the external globus pallidus (GPe) and STN.^8^, ^13^ Furthermore, ERNA was shown to align with rhythmic inhibitory synaptic input to STN from prototypic GPe neurons with ERNA amplitude being positively correlated with the potency of inhibition.^14^, ^15^ GPe neurons in turn were shown to decrease firing activity during slow wave sleep, which is consistent with the lower ERNA amplitudes during NREM sleep observed here.^16^

### Practical Use of ERNA for Adaptive DBS during Sleep

Sleep‐aware adaptive DBS paradigms in which the parameters in the control algorithms are modulated based on the decoded sleep stages may be important to improve the efficacy of DBS for nocturnal symptoms of PD. However, a reliable biomarker for sleep is required before this approach can be implemented. Spectral features in EEGs or LFPs, such as β activity, change inconsistently during sleep complicating their use for sleep stage decoding.^17^ They are also subject to movement artefacts, stimulation artefacts, and large cross‐patient variations. In comparison, ERNA comes with the advantage of high signal‐to‐noise ratio, robustness against movement and stimulation artefacts, and computational simplicity because it does not rely on real‐time time‐frequency decomposition.

### Limitations and Future Work

Results reported here are recorded when patients are *on* dopaminergic medication during acute lead externalization, it is not fully known how ERNA modulates with sleep during the *off* state and in chronically implanted patients. Additionally, the sleep recordings are brief and may be more accurately characterized as daytime naps. There was also not enough data to investigate if we can differentiate REM from NREM or awake. Further limitations include the small sample size and slight variations in the recording setup. Future studies with whole night sleep could investigate the effect of other processes on ERNA characteristics, such as the circadian rhythm, or REM sleep.

## Financial Disclosures

H.T., A.P., C.W., T.G.S, S.H., F.R.P, L.W., S.Y. were supported by the Medical Research Council (MC_UU_0003/2, MR/V00655X/1, MR/P012272/1), the MLSTF from the University of Oxford, the NIHR Oxford BRC, and the Rosetrees Trust, UK. S.H. is supported by a Brain Non‐clinical Postdoctoral Fellowship. E.A.P. has received speaking honoraria from Boston Scientific and research support from NIHR, UK Research and Innovation (UKRI), Life after Paralysis and Rosetrees Trust. F.M. has received speaking honoraria from AbbVie, Medtronic, Boston Scientific, Bial, and Merz; travel grants from the International Parkinson's Disease and Movement Disorder Society; advisory board fees from AbbVie, Merz, and Boston Scientific; consultancy fees from Boston Scientific, Merz, and Bial; research support from NIHR, UKRI, Boston Scientific, Merz, and Global Kynetic; royalties for the book *Disorders of Movement* from Springer and is a member of the editorial board of *Movement Disorders*, *Movement Disorders Clinical Practice*, and the *European Journal of Neurology*. R.S. was supported by the NIHR CL award (CL‐2021‐16‐1502). None of the other authors have any financial disclosures.

## Author Roles

(1) ResearchProject: A. Conception, B. Organization, C. Execution; (2) StatisticalAnalysis: A. Design, B. Execution, C. Review and Critique; (3) Manuscript Preparation: A. Writing of the First Draft, B. Review and Critique.

C.W.:1A, 1B, 1C, 2A, 2B, 2C, 3A, 3B

T.G.S:1B, 1C, 2A, 2B, 2C, 3B

Al.P.:1A, 1B, 1C, 2A, 2B, 2C, 3B

H.H:1C

S.H.:1C, 3B

F.R.P:1C

L.W.:1C

S.Y.:1C

X.G.:1C

R.S.:1C

A.M.:1C

An.P.:1C

A.R.:1C

A.O'K.:1C

M.G.H.:1C, 3B

F.M.:1C, 3B

E.A.P.:1C, 3B

K.A.:1C, 3B

H.T.:1A, 1B, 1C, 2A, 2B, 2C, 3B

## Supporting information


**Data S1.** Supporting Information.

**Figure S1.** Classification performance. (A) N2/N3 NREM vs wake classification performance shown for patients 1 (using single ERNA events ~2–2.5 s, and 10 consecutive events which equals ~25 seconds), 3 and 4. (B) Averages for the 5‐fold cross validation are shown for each feature.

**Table S1.** Patient information, recording details and classifier performance. UPDRS: Unified Parkinson's Disease Rating Scale; DBS: deep brain stimulation; STN: subthalamic nucleus; R: right; L: left; SG: St. George's Hospital; K: King's College Hospital; Medt: Medtronic; Boston: Boston Scientific; PRKN mut: heterozygous PRKN mutation.

**Video S1.** Patient is snoring and clearly asleep. ERNA is observed with relatively consistent low amplitude. The researchers wakethe patient up, this can be heard as the snoring stops. As the patient wakes up, the ERNA amplitude increases significantly. There is a very clear difference between ERNA amplitude while asleep and while awake.

## Data Availability

The data that support the findings of this study are openly available on the MRC BNDU Data Sharing Platform at https://data.mrc.ox.ac.uk/.
