# Supplementary material for: Stimulation‐Evoked Resonant Neural Activity in the Subthalamic Nucleus Is Modulated by Sleep
Source: Mov Disord. 2024 Nov 19;40(2):351–6. doi: 10.1002/mds.30063 (PMC11832792; doi:10.1002/mds.30063)
Supplement: Supplementary file 1 — Data S1. Supporting Information. [file MDS-40-351-s002.docx]

1. Signal processing: ERNA analysis

Continuous LFP signals were high-pass filtered at 1Hz and ERNA was analysed from the contact that showed the largest ERNA amplitude^1^. We selected a period of 40ms following every DBS pulse, as this was shown to cover the duration of ERNA in previous studies. Data within 1.2 ms after the positive deflection of the DBS pulse was removed from analysis, as this was contaminated by artefact. Subsequently, we linearly detrended the selected signal, applied a second-order low-pass filter at 700Hz and upsampled the signal using spline interpolation by a factor of 10. Different features of the ERNA were extracted after each stimulation pulse: ERNA amplitude was defined as the difference between the first peak and the next trough, latency as the time between the first peak and the peak of the stimulation pulse, width as the half-prominence width of the first peak and duration as the time between the last peak with a minimum width of 0.5 ms and minimum prominence of 10 µV, and the peak of the previous pulse^2^ (**Figure 1A**).

1. Signal processing: sleep stage classification

We trained simple linear models (support vector machine and logistic regression) for each ERNA and spectral feature for each participant to distinguish N2/N3 NREM from wakefulness. Models were trained using parameters of single ERNA events after each stimulation pulse in patients 1, 3 and 4, as well as parameters over a moving average of ten ERNA events (10 stimulation pulses) in patient 1. Ideally an average over 10 pulses would be used for all patients but there was not enough data to do this in patients 3 and 4. For spectral features, 2s prior to the stimulation pulse was averaged with 2s post pulse to find the overall spectral power. These were combined as they were found to be highly correlated on average (*r = 0.85, p<0.001*) according to the Spearman correlation. The train set was oversampled to balance the data and avoid biasing. 5-fold cross-validation was utilised to evaluate the performance of each model, while the outputs from YASA are used as a gold-standard. Classification metrics are reported in **Supplementary** **Figure 1**.

To train the multiple feature models (Best ERNA and Best Spectral in **Supplementary Figure 1**) we selected some of the best performing individual features and tested combinations of these features. These same combinations were also reduced to a single feature using tSNE, which helped to deal with correlations in the features and offered an alternative approach. From this process, only the best performing model is reported.

1. Signal processing: hypnograms

To increase the accuracy of labelling, we conducted sleep stage categorisation twice with different EEG channels (Cz-Fz and Pz for patients 1 and 2; Cz and CPz for patients 3 and 4). To mitigate for the lack of ground truth, epochs were only considered for further analysis if the classification was consistent when different EEG channels were used, and the decoding probability of the YASA model exceeded 50% in both cases (in 1 of the 4 possible outputs: awake, N1, N2/N3, or REM). Patient 2 was excluded from this final stage of the analysis (sleep stage classification) as this threshold was not met to reliably decode N2/N3 states based on EEG measurements and YASA.

Supplementary Methods References

1. Wiest C, He S, Duchet B, et al. Evoked resonant neural activity in subthalamic local field potentials reflects basal ganglia network dynamics. Neurobiol Dis 2023;178:106019.

2. Sinclair NC, McDermott HJ, Fallon JB, et al. Deep brain stimulation for Parkinson’s disease modulates high-frequency evoked and spontaneous neural activity. Neurobiol Dis 2019;130:104522.
