# Supplementary figures and images for: Stimulation‐Evoked Resonant Neural Activity in the Subthalamic Nucleus Is Modulated by Sleep
Source: Mov Disord. 2024 Nov 19;40(2):351–6. doi: 10.1002/mds.30063 (PMC11832792; doi:10.1002/mds.30063)

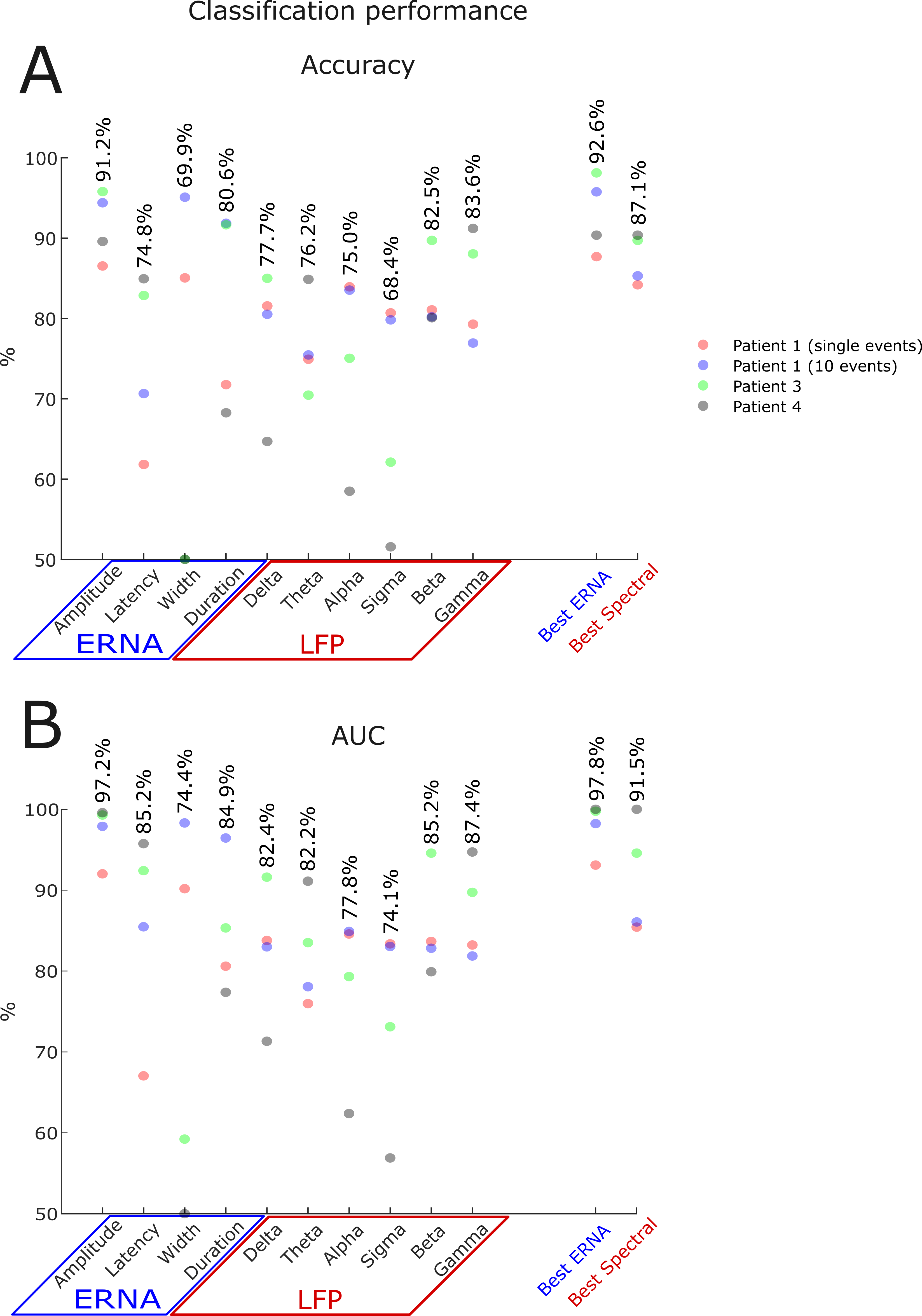

Supplement: Supplementary file 2 — Figure S1. Classification performance. (A) N2/N3 NREM vs wake classification performance shown for patients 1 (using single ERNA events ~2–2.5 s, and 10 consecutive events which equals ~25 seconds), 3 and 4. (B) Averages for the 5‐fold cross validation are shown for each feature. [file MDS-40-351-s001.tiff]
