# Supplementary material for: Stimulation‐Evoked Resonant Neural Activity in the Subthalamic Nucleus Is Modulated by Sleep
Source: Mov Disord. 2024 Nov 19;40(2):351–6. doi: 10.1002/mds.30063 (PMC11832792; doi:10.1002/mds.30063)
Supplement: Supplementary file 3 — Table S1. Patient information, recording details and classifier performance. UPDRS: Unified Parkinson's Disease Rating Scale; DBS: deep brain stimulation; STN: subthalamic nucleus; R: right; L: left; SG: St. George's Hospital; K: King's College Hospital; Medt: Medtronic; Boston: Boston Scientific; PRKN mut: heterozygous PRKN mutation. [file MDS-40-351-s003.docx]

**Supplementary Table 1.** Patient information, recording details and classifier performance. UPDRS: Unified Parkinson’s Disease Rating Scale; DBS: deep brain stimulation; STN: subthalamic nucleus; R: right; L: left; SG: St. George’s Hospital; K: King’s College Hospital; Medt: Medtronic; Boston: Boston Scientific; PRKN mut: heterozygous PRKN mutation.
